# Novel Tick-Borne Anaplasmataceae Genotypes in Tropical Birds from the Brazilian Pantanal Wetland

**DOI:** 10.3390/microorganisms12050962

**Published:** 2024-05-10

**Authors:** Amir Salvador Alabí Córdova, Alan Fecchio, Ana Cláudia Calchi, Clara Morato Dias, Anna Claudia Baumel Mongruel, Lorena Freitas das Neves, Daniel Antonio Braga Lee, Rosangela Zacarias Machado, Marcos Rogério André

**Affiliations:** 1 Vector-Borne Bioagents Laboratory (VBBL), Department of Pathology, Reproduction and One Health, School of Agricultural and Veterinarian Sciences, Sao Paulo State University “Júlio de Mesquita Filho” (FCAV/UNESP), Jaboticabal 14884-900, Brazil; amir.aiabi@unesp.br (A.S.A.C.); ana.calchi@unesp.br (A.C.C.); clara.morato@unesp.br (C.M.D.); anna.mongruel@unesp.br (A.C.B.M.); lorena.f.neves@unesp.br (L.F.d.N.); dab.lee@unesp.br (D.A.B.L.); rz.machado@unesp.br (R.Z.M.); 2 Department of Ornithology, Academy of Natural Sciences of Drexel University, Philadelphia, PA 19103, USA; alanfecchio@gmail.com

**Keywords:** *Ehrlichia* spp., *Anaplasma* spp., *Candidatus* Allocrytoplasma spp., avian hosts, South America, tick-borne diseases

## Abstract

Despite numerous reports of Anaplasmataceae agents in mammals worldwide, few studies have investigated their occurrence in birds. The present study aimed to investigate the occurrence and molecular identity of Anaplasmataceae agents in birds from the Pantanal wetland, Brazil. Blood samples were collected from 93 different species. After DNA extraction, samples positive for the avian β-actin gene were subjected to both a multiplex quantitative real-time (q)PCR for *Anaplasma* and *Ehrlichia* targeting the *groEL* gene and to a conventional PCR for Anaplasmataceae agents targeting the 16S rRNA gene. As a result, 37 (7.4%) birds were positive for *Anaplasma* spp. and 4 (0.8%) for *Ehrlichia* spp. in the qPCR assay; additionally, 13 (2.6%) were positive for Anaplasmataceae agents in the PCR targeting the 16S rRNA gene. The *Ehrlichia* 16S rRNA sequences detected in *Arundinicola leucocephala*, *Ramphocelus carbo*, and *Elaenia albiceps* were positioned closely to *Ehrlichia* sp. Magellanica. *Ehrlichia dsb* sequences detected in *Agelasticus cyanopus* and *Basileuterus flaveolus* grouped with *Ehrlichia minasensis*. The 16S rRNA genotypes detected in *Crax fasciolata, Pitangus sulphuratus* and *Furnarius leucopus* grouped with *Candidatus* Allocryptoplasma. The 23S-5S genotypes detected in *C. fasciolata*, *Basileuterus flaveolus*, and *Saltator coerulescens* were related to *Anaplasma phagocytophilum*. In conclusion, novel genotypes of *Anaplasma*, *Ehrlichia*, and *Candidatus* Allocryptoplasma were detected in birds from the Pantanal wetland.

## 1. Introduction

Anaplasmataceae agents, which comprise intracellular obligate α-proteobacteria that infect mainly the blood and endothelial cells of vertebrates, stand out amongst the vector-borne agents due to their global distribution [1,2]. The Anaplasmataceae family is composed by four genera (*Anaplasma*, *Ehrlichia*, *Wolbachia*, and *Neorickettsia*) and four putative *Candidatus* (*Candidatus* Neoerhlichia, *Candidatus* Allocryptoplasma, *Candidatus* Xenohaliotis, and *Candidatus* Xenolissoclinum) [2,3,4,5,6]. There are several reports describing different *Ehrlichia* species capable of infecting humans, namely *Ehrlichia chaffeensis* [7,8], *Ehrlichia ewingii*, *Ehrlichia muris muris*, *Ehrlichia muris eauclairensis*, *Ehrlichia canis*, and *Ehrlichia ruminantium* [2,7]. Regarding *Anaplasma* species, *Anaplasma phagocytophilum* [9], *Anaplasma platys*, *Anaplasma ovis*, and *Anaplasma capra* [7] are capable of causing illness in humans. *Neorickettsia sennetsu* [10] and *Candidatus* Neoehrlichia mikurensis [11] are also known to infect humans. This broad capability to infect humans has made them of great importance to public health [12,13].

Despite several reports of these agents in mammals around the world [14], few studies have investigated the occurrence of Anaplasmataceae agents in birds. For instance, *Anaplasma phagocytophilum* has been detected in *Ixodes* ticks collected from birds in Canada [15], Greece [16], Lithuania, Norway [17], France [18], and Latvia [19], emphasizing that avian hosts can disperse ticks infected with zoonotic *Anaplasma* species. When it comes to detection of these agents in birds, *A. phagocytophilum* was detected in blood samples from thrushes (*Zoothera aurea*, *Turdus cardis*, and *Turdus palidus*) in South Korea [20], in liver samples from collared doves (*Streptopelia decaocto*), and Eurasian eagle owls (*Bubo bubo*) in China [21] as well as in tissue samples (heart, liver, spleen, and kidney) in a robin (*Erithacus rubecula*) and a song thrush (*Turdus philomelos*) in Romania [22]. *Candidatus* Anaplasma sphenisci was detected in blood samples from penguins (*Spheniscus demersus*) in South Africa [23]. In Chile, a putative novel *Ehrlichia* sp. was detected in spleen samples from penguins (*Spheniscus magellanicus*) [24]. An *Ehrlichia* genotype closely related to *Ehrlichia chaffeensis* was detected in blood and spleen samples from a song thrush (*Turdus philomelos*) in Hungary [25] and in liver samples from a common pheasant (*Phasianus colchicus*) from China [21].

In Brazil, previous studies reported the occurrence of *Anaplasma* 16S rRNA genotypes closely related to *Anaplasma phagocytophilum* in blood samples from black vultures (*Coragyps atratus*), caracaras (*Caracara plancus*) [26], dusky-legged guan (*Penelope obscura*) [27], Orinoco geese (*Neochen jubata*) [28], burrowing owl (*Athene cunicularia*), tropical screech owl (*Megascops choliba*), roadside hawk (*Rupornis magnirostris*), barn owl (*Tyto alba*), striped owl (*Asio clamator*), and Aplomado falcon (*Falco femoralis*) [29]. *Ehrlichia* 16S rRNA genotypes closely related to *Ehrlichia chaffeensis* were detected in blood samples from an American kestrel (*Falco sparverius*) [26], Orinoco goose [28], burrowing owl, tropical screech owl, roadside hawk (*Rupornis magnirostris*), and barn owl (*Tyto alba*) [29]. *Ehrlichia* 16S rRNA genotypes closely related to *E. canis* were detected in blood samples from black vulture, Orinoco goose [26,28], roadside hawk, and striped owl [29].

Greater comprehension of the role of avian hosts in the eco-epidemiology of vector-borne agents [30] is needed to hamper the potential spill-over of emergent zoonotic pathogens [31]. Birds play an important role as carriers of ticks, contributing to the dispersal of zoonotic agents such as Rickettsiales agents [32] and are responsible for harboring several vector-borne agents such as Anaplasmataceae agents [20,22,25,26].

Taking into account the huge diversity of resident and migratory birds in the Pantanal wetland [33,34], the multiple ecosystems that provide preservation of the biodiversity [35], and the feasibility of the transmission of vector-borne pathogens among a diverse avian population and its influence on the genetic richness of vector-borne pathogens, the current study aimed to investigate the occurrence and genetic diversity of tick-borne Anaplasmataceae agents in birds in the Pantanal wetland in the states of Mato Grosso and Mato Grosso do Sul, central-western Brazil.

## 2. Materials and Methods

### 2.1. Study Area and Bird Sampling

**Figure 1:**
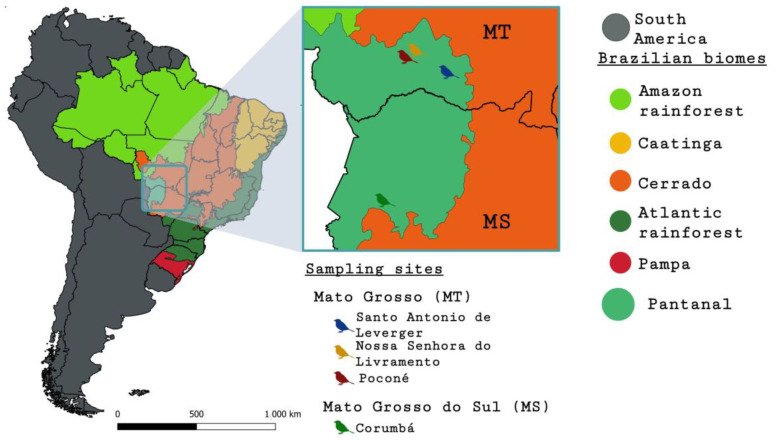
Brazilian biomes and sampling sites in the Pantanal wetland in the states of Mato Grosso and Mato Grosso do Sul.

The sampling period comprised the months of April and August through November 2019. The birds were caught with 20 mist nets with dimensions of 36 mm mesh, 12 m long and 2.5 m in height, allocated next to the tracks in four sampling sites in the region of Pantanal, namely Nossa Senhora do Livramento (99 samples) (16°21′46.8″ S 56°17′24.0″ W), Poconé (100 samples) (16°29′56.4″ S 56°24′46.8″ W), and Santo Antonio de Leverger (200 samples) (16°44′34.8″ S 55°33′10.8″ W) from the state of Mato Grosso and Corumbá (101 samples) (19°34′37.2″ S 57°01′08.4″ W) from the state of Mato Grosso do Sul (Figure 1). In each locality, the sampling was performed over a five-day period. The mist nets were opened after dawn and checked periodically every 30 min.

Bird species were identified as previously mentioned [36]. A total of 517 blood samples were collected from the following orders: 1 Accipitriformes, 4 Apodiformes, 3 Charadriiformes, 14 Columbiformes, 12 Coraciiformes, 12 Cuculiformes, 1 Galliformes, 2 Gruiformes, 452 Passeriformes, 4 Pelecaniformes, 5 Piciformes, 5 Psittaciformes, and 1 Tinamiformes. The samples are from a previous study [36] (Supplementary Table S1).

The identification of birds was performed by an skilled ornithologist using several field guides. Bird taxonomy follows the Birdtree project (see https://birdtree.org/taxonomy) (accessed on 10 April 2024) [37]. Representative bird specimens were deposited in the Bird Collection of UFMT, Cuiabá, Brazil. All birds and blood samples were collected under appropriate permits in Brazil.

Sampling procedures involving wild birds were approved by IBAMA (72548 e 72790), the “Comissão de Ética no Uso de Animais” of the Faculdade de Ciências Agrárias e Veterinárias (FCAV/UNESP) (CEUA 268/21) and SISGEN (AF30FD1).

### 2.2. Molecular Assays

#### 2.2.1. DNA Extraction and PCR for Avian Endogenous Gene (*β-Actin*)

DNA extraction and PCR for avian *β-actin* as described by Hatai et al. [38] were previously processed [36] (Supplementary Table S2).

#### 2.2.2. Molecular Screening for *Anaplasma* spp. and *Ehrlichia* spp. Targeting the *groEL* Gene by a Multiplex Quantitative Real-Time (q)PCR

DNA avian blood samples that were positive for the endogenous avian β-actin gene were then screened for *Anaplasma* spp. and *Ehrlichia* spp. using a multiplex qPCR targeting the *groEL* gene, as previously described [39]. The reactions consisted of 1 μL of sample DNA, 0.2 μM of each oligonucleotide primer and hydrolysis probe, Master Mix 2x (GoTaq ™ Probe qPCR Master Mix, Promega Corporation, Madison, WI, USA), and sterilized ultra-pure water (Nuclease-Free Water, Promega^®^, Madison, WI, USA) to complete a final volume of 10 μL. Amplification reactions were carried out in a CFX96 Thermal Cycler (BioRad^®^, Hercules, CA, USA) (Supplementary Table S2). Samples were tested in duplicates.

Quantification of the number of target DNA copies/μL was performed using G-Blocks (G-Blocks, Integrated DNA Technologies^®^, Coralville, IA, USA) containing the target sequences. Serial dilutions were made to construct standards with different concentrations of G-Blocks DNA containing the target sequence (2.0 × 10^7^ copies/μL to 2.0 × 10^0^ copies/μL) in order to obtain the efficiency and correlation coefficient of reactions. The number of target gene copies was determined according to the formula (Xg/μL DNA/(plasmid size (bp) × 660)) × 6.022 ×1023 × plasmid copies/μL. All analyses were carried out in accordance with standards established by MIQE (“Minimum Information for Publication of Quantitative real-time PCR Experiments”) [40]. Quantification cycles (Cq) with a difference of no more 0.5 were considered positive; otherwise, they were repeated in triplicates. Cq is considered as the fraction of a PCR cycle where the target in the samples is quantified [41].

#### 2.2.3. Molecular Screening for Anaplasmataceae Agents Targeting the 16S rRNA Gene by Conventional PCR

Avian DNA samples that were positive for the endogenous avian *β-actin* gene were also screened for Anaplasmataceae agents using the previously described PCR targeting the 16S rRNA gene [42]. Oligonucleotides sequences and thermal conditions are shown in Supplementary Table S2.

#### 2.2.4. PCR Assays for Molecular Characterization

Avian DNA samples that were positive for the 16S rRNA gene for Anaplasmataceae agents in the screening PCR [42] and/or for the *groEL* gene in the multiplex qPCR for *Anaplasma* spp. and *Ehrlichia* spp. [39] were subjected to an additional previously described conventional PCR assays for *Ehrlichia* spp. based on the *dsb* [43], *gltA* [44,45], *sodB* [46], *omp*-1 [47], *rpoB*, *groEL*, and *ftsZ* [44,48] genes.

For *Anaplasma* molecular characterization, positive samples in the screening PCR assays were subjected to additional previously described conventional PCR assays based on the 16S rRNA gene [20,42,49,50,51,52,53], and the intergenic fragment 23S-5S (ITS) [54], as well as to a nested PCR based on the *groEL* gene [55]. Samples in which sequences related to *Candidatus* Allocryptoplasma spp. were detected were subjected to additional previously described conventional PCR assays targeting the *groEL* and *sucA* genes [4]. The primer oligonucleotides and thermal sequences used in PCR assays are presented in Supplementary Table S2.

Each reaction had a total volume of 25 µL: 1.25 U Go Taq Hot Start Polymerase (Promega^®^, Madison, WI, USA), PCR buffer 10× (Promega^®^, Madison, WI, USA), sterilized ultra-pure water (Invitrogen^®^, Carlsbad, CA, USA), 0.2 mM of each deoxynucleotide, 0.4 µM of each oligonucleotide, 3.0 mM of MgCl_2_, and 5 µL DNA template.

*Ehrlichia canis* (Jaboticabal strain) DNA (obtained from DH82-infected cells [56]) and *Anaplasma* sp. DNA (obtained from cattle from Mozambique [57]) were used as positive controls in all conventional and nested PCR assays for *Ehrlichia* spp. and *Anaplasma* spp., respectively. Sterilized ultra-pure water (Invitrogen^®^, Carlsbad, CA, USA) was used as a negative control in all PCR assays.

### 2.3. Gel Electrophoresis and Amplicon Purification

PCR products were separated in a 1% agarose gel stained with ethidium bromide (0.5 μL/mL) in TBE buffer (pH 8). A molecular-weight size marker of 100 pb was employed to verify the size of the PCR products (Kasvi, Campina, São José dos Pinhais, PR, Brazil). Agarose gels were visualized under an ultra-violet transilluminator Chemidoc (BioRad^®^, Hercules, CA, USA) and image analyzer software, Image Lab (BioRad^®,^ Hercules, CA, USA). Amplicons were purified with the Wizard SV Gel and PCR cleanup system kit (Promega, Madison, WI, USA), following manufacturer’s recommendations.

### 2.4. Sequencing and nBLAST Analysis

After purification, PCR products were submitted to sequencing by dideoxynucleotide chain termination method [58]. Sequencing was performed in an ABI PRISM 3700 DNA Analyzer (Applied Biosystems, Waltham, MA, USA) at “Centro de Recursos Biológicos e Biologia Genômica” (CREBIO -FCAV -UNESP, Jaboticabal, SP, Brazil).

The retrieved sequences were trimmed, and the consensus sequences were assembled utilizing BioEdit v7.2.5 software [59]. The consensus sequences were compared in nBLAST [60] with homologous sequences deposited in the GenBank database (http://www.ncbi.nlm.nih.gov/genbank) (accessed on 12 April 2024) [61].

Sequences can be accessed by the following accession numbers: PP326052, PP326053, PP326054, PP346669, PP346773, PP373675, PP373692, PP373702, PP417932, PP417933, PP417934, PP417935, and PP417936.

### 2.5. Phylogenetic Analyses

The obtained sequences were aligned with homologous sequences obtained from the Genbank database, using the Clustal/W software v. 1.81 [62] via Bioedit v. 7.0.5.3 [59]. Alignments saved in “FASTA” mode were used to infer the evolutionary model and the maximum likelihood analyses performed using the online version of IQtree version (http://iqtree.cibiv.univie.ac.at/) (accessed on 27 March 2024) [63]. Clade supports for maximum likelihood (ML) analyses were assessed using bootstrap analyzes [64] of 100 repetitions. Editing of phylogenetic trees as well as the outgroup was performed using Figtree V1.4.4 software (http://tree.bio.ed.ac.uk/software/figtree/) (accessed on 7 April 2024) [65].

## 3. Results

### 3.1. Molecular Screening for Endogenous Gene Avian ß-Actin

The values of mean concentration and 260/280 ratio of the DNA-extracted samples can be found in a previously published work [36]. Individual DNA samples’ ratios and concentrations were estimated, and those samples that exceeded 100 ng/µL were diluted to a final concentration 50 ng/µL with sterilized ultra-pure water (Invitrogen^®^, Carlsbad, CA, USA). The 17 samples negative for avian β-actin gene were #13 (SL068) *Coccycua minuta*, #412 (SL48) *Picumnus albosquamatus*, #500 (SL55) *P. albosquamatus*, and #505 (SL56) *Hemitriccus striaticollis* from Santo Antonio de Leverger; #99 (BAP048) *Ramphocelus carbo*, #138 (BEP259) *Donacobius atricapilla*, #146 (BEP268) *D. atricapilla*, #257 (BAP52) *Cacicus cela*, #269 (BAP85) *C. cela*, #295 (BAP33) *Cnemotriccus fuscatus*, and #296 (BAP70) *Cercomacra melanaria* from Poconé; #308 (BAP144) *Aramides cajanea*, #364 (BAP187) *P*. *albosquamatus*, #385 (BAP266) *P. albosquamatus*, and #390 (BAP308) *Thraupis palmarum* from Nossa Senhora do Livramento; and #96 (BEP424) *Cyanocorax cyanomelas*, #99 (BAP048) *Ramphocelus carbo*, #138 (BEP259) *D. atricapilla*, and #146 (BEP268) *D. atricapilla* from Corumbá.

### 3.2. Molecular Screening for Anaplasma spp. and Ehrlichia spp. by a Quantitative Real-Time PCR Based on the groEL Gene

Out of 500 avian DNA samples that were submitted to a multiplex qPCR for *Anaplasma* spp. and *Ehrlichia* spp. based on the *groEL* gene, 37 (7.4%) were positive for *Anaplasma* spp., and 4 (0.8%) were positive for *Ehrlichia* spp. Co-positivity for *Anaplasma* spp. and *Ehrlichia* spp. was found in two samples (0.4%) (Supplementary Table S3). Positivity for *Anaplasma* spp. by avian species was 2/14 (14.28%) *Agelasticus cyanopus*, 1/20 (5%) *Basileuterus flaveolus*, 1/1 (100%) *Busarellus nigricollis*, 1/9 (11.11%) *Cacicus cela*, 1/5 (20%) *Cantorchilus leucotis*, 1/12 (8.33%) *Certhiaxis cinnamomeus*, 1/5 (20%) *Chloroceryle americana*, 1/5 (20%) *Cranioleuca vulpina*, 1/4 (25%) *Dendroplex picus*, 1/6 (16.66%) *Eucometis penicillata*, 1/2 (50%) *Guira guira*, 1/3 (33.33%) *Icterus cayanensis*, 1/4 (25%) *Legatus leucophaius*, 3/10 (30%) *Leptotila verreauxi*, 1/26 (3.84%) *Paroaria capitata*, 1/1 (100%) *Phaetornis nattereri*, 1/7 (14.28%) *Pipra fasciicauda*, 2/21 (9.52%) *P. sulphuratus*, 9/73 (12.32%) *R*. *carbo*, 1/16 (6.25%) *S*. *coerulescens*, 1/8 (12.5%) *Sporophila angolensis*, 1/5 (20%) *Sporophila collaris*, 1/3 (33.33%) *Thraupis sayaca*, 1/3 (33.33%) *Tigrisoma lineatum*, and 1/5 (20%) *Tyrannus melancholicus.* Positivity for *Ehrlichia* spp. by avian species was 1/12 (8.33%) *C. cinnamomeus,* 1/4 (25%) *D. picus* (coinfection with *Anaplasma* sp.), 1/16 (6.25%) *Furnarius rufus*, and 1/73 (1.36%) *R. carbo* (coinfection with *Anaplasma* sp.). According to locality, the higher positivity was found among birds in Poconé, with 30/100 (30%) birds found positive, followed by the locality of Nossa Senhora do Livramento with 12/99 (12.1%) infected birds and Santo Antonio de Leverger with 15/200 (7.5%) infected birds, while the locality which presented the lowest infection frequency was Corumbá with 3/101 (3%) birds.

While the Cq values of *Anaplasma*-positive samples ranged from 28.94 to 39.24, *Ehrlichia*-positive samples showed Cq values ranging from 33.24 to 37.89. Even after re-testing the positive samples in triplicates, achievement of the estimated quantification was possible in only one positive sample for *Anaplasma* spp. probably due to Monte Carlo effect [40]: BAP87 (232) from a specimen of *Legatus leucophaius* from Poconé, with an average Cq of 37.96 and an average quantification of 5.845 × 10^−1^ copies/ µL. All samples positive in the qPCR were negative in additional conventional PCR assays based on the 16S rRNA, *dsb*, *gltA*, *sodB*, *omp*-1, *rpoB*, *groEL*, and *ftsZ* genes and 23S-5S (ITS) intergenic region, mostly likely due the low bacteremia at the time of blood sampling.

### 3.3. Molecular Screening for Anaplasmataceae Agents by a cPCR Based on the 16S rRNA Gene and Molecular Characterization

Thirteen avian DNA samples were positive in the screening for Anaplasmataceae agents targeting the gene 16S rRNA (345 bp) [42]: *Ehrlichia* sp. was detected in one *R. carbo* from Poconé (MT), *Ehrlichia* sp. was detected in one *E. albiceps* from Poconé (MT), and *Ehrlichia* sp. was detected in one *R. carbo* from Santo Antonio de Leverger (MT). *Anaplasma* sp. was detected in one *P. sulphuratus* from Santo Antonio de Leverger (MT). *Candidatus* Allocryptoplasma spp. was detected in one *C. fasciolata* from Nossa Senhora do Livramento (MT). The BLAST results regarding the five 16S rRNA sequences (ranging from 179–281 bp) obtained in the Parola et al. (2000)’s protocol [42] are shown in Supplementary Table S4.

When combining the results obtained by the multiplex qPCR (*groEL*) and cPCR (16S rRNA gene) assays and comparing the positivity for *Anaplasma* spp./*Candidatus* Allocryptoplasma spp. and *Ehrlichia* spp., a higher positivity for *Anaplasma* spp./*Candidatus* Allocryptoplasma spp. (9.7%; 37/382) and *Ehrlichia* spp. (2.3%; 9/382) was found among birds sampled in the state of Mato Grosso. On the other hand, a positivity of 2.5% (3/118) for *Anaplasma* spp. was found among birds sampled in the state of Mato Grosso do Sul.

When performing conventional PCR assays to characterize Anaplasmataceae agents with the 16S rRNA [52], *dsb* gene [43], and 23S-5S rRNA intergenic region (ITS) [54], three sequences (ranging from 832 to 884 bp) were obtained in the semi-nested PCR protocol targeting a fragment of 800 bp of the 16S rRNA gene [52]. The positive samples were obtained from one *C. fasciolata,* one *F. leucopus*, and one *A. leucocephala* from Nossa Senhora do Livramento.

Two sequences (ranging from 401 to 402 bp) were obtained in the PCR protocol targeting the *dsb* gene. The two positive samples were obtained from one *A. cyanopus* from Nossa Senhora do Livramento (MT) and one *B. flaveolus* from Santo Antonio de Leverger (MT).

Three sequences (ranging from 832 to 884 bp) were obtained in the PCR protocol targeting the 23S-5S rRNA intergenic region (ITS). The positive samples were obtained from one *C. fasciolata* from Nossa Senhora do Livramento (MT) and one *S. coerulescens* and one *B. flaveolus* from Santo Antonio de Leverger (MT). The BLAST results for the obtained 16S rRNA, *dsb*, and ITS sequences are shown in Supplementary Table S4.

Sequences showing high identity to *Candidatus* Allocryptoplasma sp. and obtained in the PCR protocols described by Parola [42] or Eberhardt [52] targeting the 16S rRNA gene were submitted to additional PCR assays based on the *sucA* and *groEL* genes [4]. As a result, one *C. fasciolata* from Nossa Senhora do Livramento (MT) showed positivity in the PCR targeting the *groEL* gene, but due to low intensity of the obtained band, it was not possible to retrieve a readable sequence.

### 3.4. Phylogenetic Analyses

**Figure 2:**
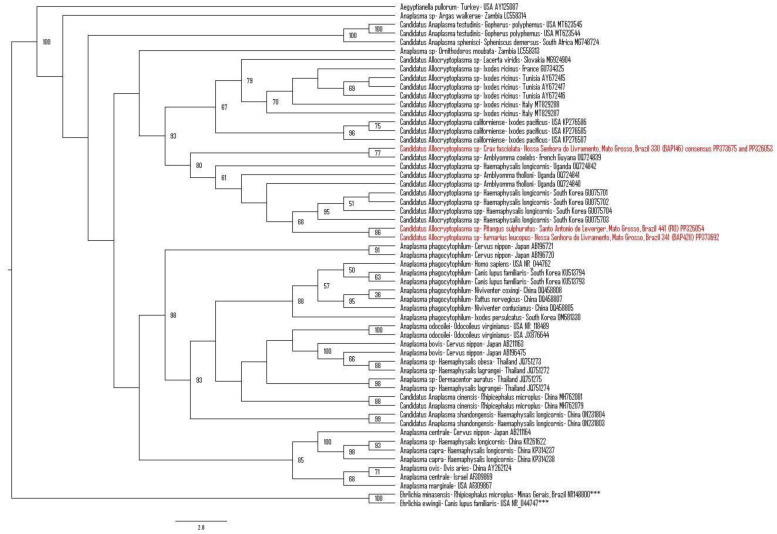
Phylogenetic analysis generated by the maximum likelihood method and TIM2+F+I+G4 evolutionary model based on an alignment of 1439 bp of 16S rRNA gene sequences from Anaplasmataceae agents, containing 57 homologous sequences for the 16S rRNA gene from the genera *Anaplasma*, *Candidatus* Allocryptoplasma sp., and *Aegyptianella* sp. *Ehrlichia minasensis* and *Ehrlichia ewingii* sequences were used as outgroups (NR148800 and NR_044747) and are indicated with (***). The sequences obtained in this project are highlighted in red. Bootstraps lower than 50 are not shown.

The phylogenetic analysis based on an alignment of 1439 bp positioned the sequences detected herein in a clade exclusively formed by *Candidatus* Allocryptoplasma spp. previously detected in *Ixodes ricinus* ticks (OQ724839) from French Guiana; in a green lizard (*Lacerta viridis*) (MG924904) from Slovakia; in *Ixodes ricinus* ticks (GU734325, AY672415, AY672416, AY672417, MT829288, and MT829287) from France, Tunisia, and Italy; in *Candidatus* Allocryptoplasma californiense (KP276585–KP276587) detected in *Ixodes pacificus* ticks from the USA; in *Candidatus Allocryptoplasma* sp. detected in *Amblyomma tholloni* ticks (OQ724840–OQ724842) from Uganda; in an *Amblyomma coelebs* tick (OQ724839) from French Guiana, and in *Haemaphysalis longicornis* ticks from South Korea (GU075701–GU075704). The consensus sequence of *Candidatus* Allocryptoplasma spp. detected in a *Crax fasciolata* from Nossa Senhora do Livramento, Mato Grosso (#330 BAP146) (PP373675 and PP326053) was closely related to the sequence of *Candidatus* Allocryptoplasma sp. detected in an *Amblyomma coelebs* tick (OQ724839) from French Guiana, while the sequences detected in *P. sulphuratus (*#441 (F10)) (PP326054) from Santo Antonio de Leverger and in a *F. leucopus* (#341 BAP428) (PP373692) from Nossa Senhora do Livramento were closely related to *Candidatus* Allocryptoplasma sp. sequences detected in *Haemaphysalis longicornis* from South Korea (GU075701–GU075704) (Figure 2).

**Figure 3:**
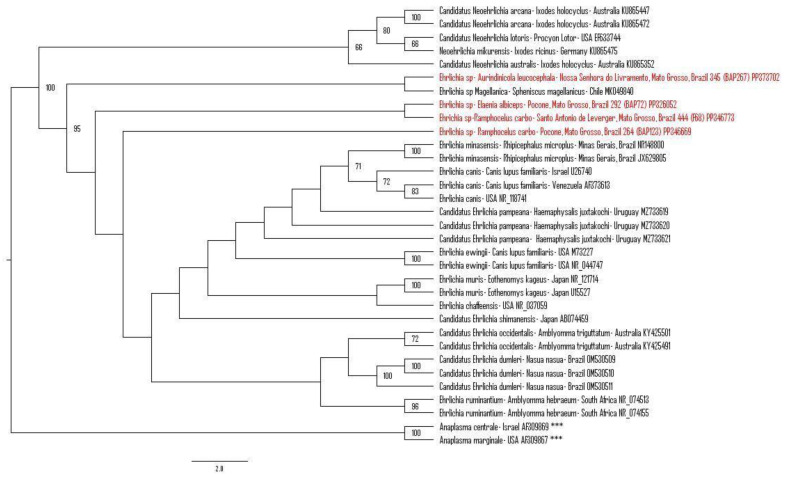
Phylogenetic analysis generated by the maximum likelihood method and GTR+I+G4+F evolutionary model based on an alignment of 1339 bp of 16S rRNA gene sequences from *Ehrlichia* spp. and *Neoehrlichia* spp., containing 33 homologous sequences. *Anaplasma centrale* (AF309869) and *Anaplasma marginale* (AF309867) sequences were used as outgroups and are indicated with (***). The sequences obtained in this project are highlighted in red. Bootstraps lower than 50 are not shown.

The phylogenetic analysis based on an alignment of 1339 bp positioned the obtained sequences in three different clades. The first clade grouped the *Ehrlichia* sequence obtained from an *Aurindinicola leucocephala* from Nossa Senhora do Livramento, Mato Grosso, Brazil (#345 BAP267) (PP373702), with *Ehrlichia* sp. Magellanica detected in a Magellanic penguin (*Spheniscus magellanicus*) from Chile (MK049840). The second clade was exclusively formed by sequences detected in the present study and were obtained from an *Elaenia albiceps* from Poconé, Mato Grosso, Brazil (#292 BAP72) (PP326052), and a *Ramphocelus carbo* from Santo Antonio de Leverger (#444 F68) (PP346773). The third clade was formed by a sequence of *Ehrlichia* sp. detected in an *R. carbo* from Poconé, Mato Grosso, Brazil (#264 BAP123) (PP346669) (Figure 3).

**Figure 4:**
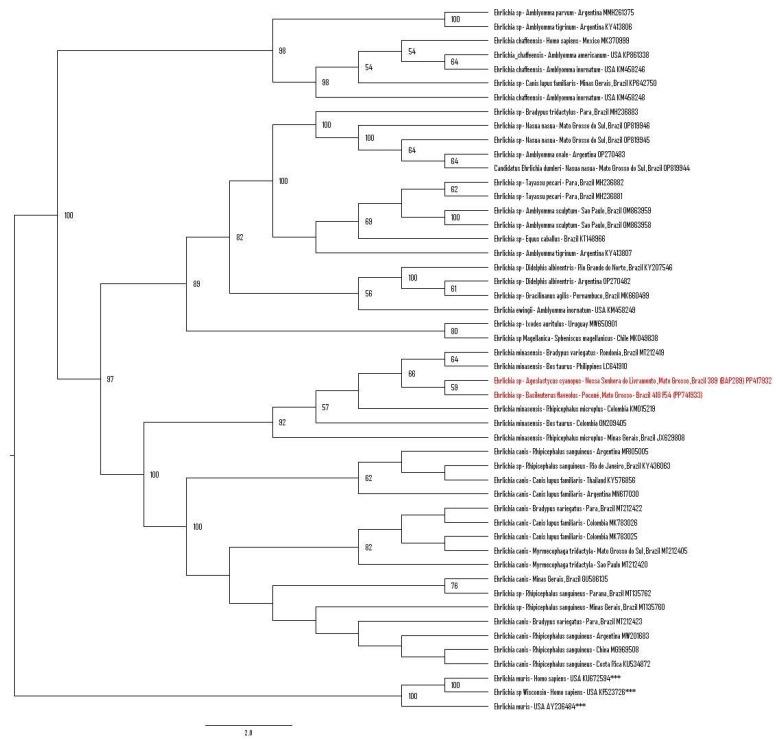
Phylogenetic analysis generated by the maximum likelihood method and TIM3+I+G4+F evolutionary model based on an alignment of 409 bp of *dsb* gene sequences, containing 53 homologous sequences. *Ehrlichia muris* and *Ehrlichia* sp. were used as outgroups (KU672594, KF523726, and AY236484) and are indicated with (***). The sequences obtained in this project are highlighted in red. Bootstraps lower than 50 are not shown.

The phylogenetic analysis based on an alignment of 409 bp of the *dsb* gene positioned the two sequences obtained in the present study into the same clade: The sequences obtained from *A. cyanopus* (#389 (BAP289)) (PP417932) from Nossa Senhora do Livramento and from *B. flaveolus* (#418 (F54)) (PP417933) from Poconé were closely related to *Ehrlichia minasensis* obtained from a common sloth (*Bradypus variegatus*) (MH212419) from Brazil, from cattle from the Philippines (LC641910) and Colombia (ON209405), from *Rhipicephalus microplus* ticks from Brazil (JX629808) and Colombia (KM015219), and from a *Rhipicephalus sanguineus* tick from Brazil (MT135769) (Figure 4).

**Figure 5:**
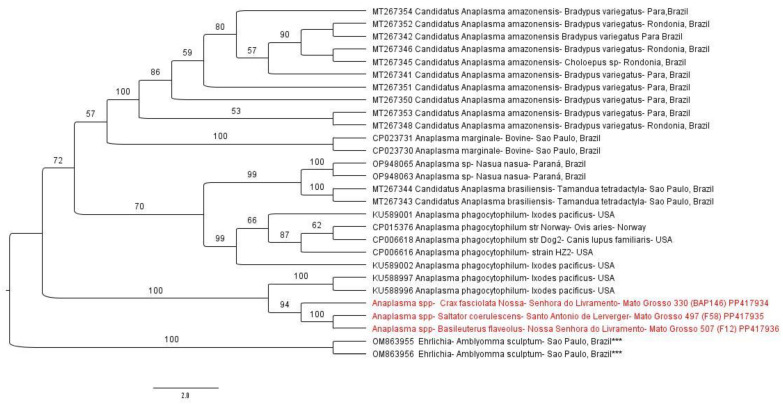
Phylogenetic analysis generated by the maximum likelihood method and evolutionary model TVM+G4+I+F based on an alignment of 430 bp of sequences from the 23S-5S intergenic region of *Anaplasma* sp., containing 26 homologous sequences from the 23S-5S intergenic region from *Anaplasma* sp. Two sequences from *Ehrlichia* spp. used as outgroups (OM863955 and OM863956) are indicated with (***). The sequences obtained in this project are highlighted in red. Bootstraps lower than 50 are not shown.

The phylogenetic analysis based on an alignment of 430 bp of the 23S-5S intergenic region (ITS) positioned the sequences obtained herein (*C. fasciolata* (#330 BAP146) (PP417934) and *B. flaveolus* (#507 F12) (PP417936) from Nossa Senhora do Livramento and *S. coerulescens* (#497 F58) (PP417935) from Santo Antonio de Leverger) in the same clade, grouping with sequences of *A. phagocytophilum* (KU588997; KU588996) from *Ixodes pacificus* ticks from the USA (Figure 5).

## 4. Discussion

Although Anaplasmataceae agents have been described in wild mammals worldwide [14], studies focusing on the occurrence and genetic diversity of these tick-borne α-proteobacteria in avian hosts are scarce. Up to now, these agents have been molecularly detected in avian hosts from South Africa [23], Chile [24], South Korea [20], Hungary [25], Romania [22], Cyprus [66], and Brazil [26,27,28,29].

In the present study, the molecular occurrence of *Ehrlichia/Anaplasma* in birds inferred by both conventional (16S rRNA) and real-time (*groEL*) PCR assays used as screening tests was 9.2% (46/500), falling within previously reported prevalence in birds, which ranges from 0.73% [25] up to 43.5% [28]. Such difference in molecular prevalence for *Ehrlichia/Anaplasma* in birds among different studies might be due to the variety of avian species tested, the sensitivity/specificity of PCR protocols used, geographic region, level of infestation by tick vectors, and susceptibility of the birds sampled with regard to the development of detectable bacteremia, among others.

In the present study, the *Ehrlichia* 16S rRNA sequences detected in specimens of *A. leucocephala* (#345 BAP267) (PP373702), *R. carbo* (#444 F68 (PP346773) and #264 BAP123 (PP346669)), and *E. albiceps* (#292 BAP72 (PP326052)) were positioned closely to *Ehrlichia* sp. Magellanica, detected in a Magellanic penguin (*Spheniscus magellanicus*) from Chile [24]. In the phylogeny based on the *dsb* gene, *Ehrlichia* sequences detected in specimens of *A. cyanopus* (#389) and *B. flaveolus* (#418) were grouped with *Ehrlichia minasensis,* suggesting that *Ehrlichia minasensis* or a closely related genotype might be transmitted by ticks to a variety of hosts besides cattle, such as sloths [67] and birds.

Herein, 3.7% of sampled birds were positive in the qPCR for *Anaplasma* spp. based on the *groEL* gene. Unfortunately, all samples positive in the qPCR were negative in conventional PCR assays, which is similar to the results reported by [29] among carnivorous birds from Brazil.

The phylogeny based on the 23S-5S rRNA intergenic region presented well-established clades similar to those reported by Calchi [67] and Perles [68,69]. The ITS sequences obtained in the present work in blood samples from specimens of *C. fasciolata* (#330 BAP146 (PP326053)), *B. flaveolus* (#507 F12 (PP417936)), and *S. coerulescens* (#497 F58 (PP417934)) were positioned in a clade closely related to *A. phagocytophilum*. Previously, in Brazil, 16S rRNA genotypes of *Anaplasma* sp. detected in runner geese [28], vultures, caracaras [26], and curassows [27] were positioned closely to *A*. *phagocytophilum* and *Anaplasma* sequences previously detected in wild [70] and domestic felids [71]. In South Korea, *Anaplasma* 16S rRNA sequences detected in thrushes showed to be closely related to *A*. *phagocytophilum* [20]. In South Africa, the 16S rRNA genotype of *Anaplasma* sp. detected in African penguins was phylogenetically associated with *Anaplasma capra*, *A*. *marginale*, *A*. *ovis*, and *A*. *centrale* [23]. Such phylogenetic positioning based on short fragments of the 16S rRNA should be interpreted with caution.

In the phylogenetic analysis based on the 16S rRNA gene, the genotypes detected in specimens of *C. fasciolata* (#330 BAP146 (PP326053)) and *F. leucopus* (#341 BAP428 (PP373692)) were positioned within the clade of *Candidatus* Allocryptoplasma sp. Previous studies have shown that *Candidatus* Cryptoplasma sp., recently named as *Candidatus* Allocryptoplasma sp. [4], presents itself as a monophyletic clade [4,72]. When comparing the phylogenetic tree of the present study with that one described by Ouass [4], it is possible to observe the proximity of *Candidatus* Allocryptoplasma and *Anaplasma* spp. While in the phylogeny of the present study, it was possible to observe a polyphyletic clade of *Candidatus* Allocryptoplasma, a monophyletic profile was observed for the available sequences of *Candidatus* Allocryptoplasma sp. in the studies developed by Ouass [4] and Mendoza-Roldan [72]. This discrepancy can be explained by the inclusion of distinct and novel genotypes detected in the present study, which indicates that the phylogenetic positioning of such agents is far from being resolved.

*Candidatus* Allocryptoplasma has already been reported in *Ixodes ricinus* from Morocco and Tunisia [73], *Ixodes pacificus* from the United States of America [3], *Ixodes ricinus* from Serbia [74], *Ixodes ricinus* from France [75], *Amblyomma tholloni* and *Haemaphysalis parmata* from Uganda and *Amblyomma coelebs* from French Guiana [4], *Lacerta viridis* lizards and *Apodemus agrarius* rodents from Slovakia [76], *Podarcis* lizards from Italy [71], as well as in an *Amblyomma dissimile* tick from Brazil [77]. *Candidatus* Allocryptoplasma spp. present apparent worldwide distribution, whose implications for animal and human health are still unknown. Since the majority of *Candidatus* Allocryptoplasma sequences were obtained from ticks (*Ixodes ricinus*, *Ixodes pacificus*, *Amblyoma tholloni*, *Amblyomma dissimile*, and *Haemaphysalis longicornis*) so far, it has been suggested that ticks might play an important role in the transmission of such agents to hosts. When it comes to vertebrate hosts, *Candidatus* Allocryptoplasma spp. or closely related agents have only been detected in reptiles [72,78] and rodents [76]. The present work showed, for the first time, the occurrence of *Candidatus* Allocryptoplasma spp. or a closely related agent in birds. Altogether, these findings emphasize the need to unravel the diversity of Anaplasmataceae agents in non-classical vertebrate hosts such as birds.

Taking into account that the birds analyzed in the present work originated from a previous study that investigated tick infestation [34], only 2/37(5.55%) birds positive for *Anaplasma* spp. were parasitized by ticks at the sampling time: *R. carbo* #417 (F073) was infested by an *Amblyomma longirostris* nymph, and *Eucometis penicillata* #51 (SL025) was found parasitized by an *Amblyomma nudosum* nymph. Future studies should be performed in order to unravel the tick species involved in the transmission of these novel Anaplasmataceae genotypes among birds.

## 5. Conclusions

Novel genotypes of *Ehrlichia* spp. (closely related to *Ehrlichia* Magellanica and *E. minasensis*), *Anaplasma* spp. (closely related to *A. phagocytophilum*), and *Candidatus* Allocryptoplasma spp. were molecularly detected in blood samples from tropical bird species sampled in the Pantanal wetland. More studies are needed to molecularly characterize and unravel the transmission dynamics of these Anaplasmataceae agents in tropical birds from Pantanal wetland in order to investigate the role of wild birds in the dispersion of such agents. Whole-genome sequencing approaches should be performed with the aim of shedding light on the real identity of these novel Anaplasmataceae agents infecting neotropical wild birds. This is the first molecular evidence of *Candidatus* Allocryptoplasma spp. in birds in the world.

## Data Availability

The sequences generated during the study were submitted to the NCBI Genbank (https://www.ncbi.nlm.nih.gov/genbank/) (accessed on 28 February 2024).

## Supplementary Materials

The following supporting information can be downloaded at: https://www.mdpi.com/article/10.3390/microorganisms12050962/s1, Table S1: Number of avian species collected in Pantanal wetland in the states of Mato Grosso and Mato Grosso do Sul, central-western Brazil. Table S2: Conventional, nested, and quantitative real-time PCR assays used in screening and molecular characterization for Anaplasmataceae agents targeting the 16S RNA, *groEL, dsb, gltA, sodB, omp-1, rpoB, ftsZ,* and *sucA* genes and intergenic region 23S-5S (ITS). Table S3: Avian DNA samples positive in the multiplex quantitative (q) real-time qPCR for *Anaplasma* spp. and *Ehrlichia* spp. based on the *groEL* gene. Table S4: BLAST analyses results obtained for 16S rRNA, *dsb*, and ITS sequences obtained in PCR protocols for Anaplasmataceae agents from avian blood samples from the Brazilian Pantanal.
