# Supplementary material for: Novel Tick-Borne Anaplasmataceae Genotypes in Tropical Birds from the Brazilian Pantanal Wetland
Source: Microorganisms. 2024 May 10;12(5):962. doi: 10.3390/microorganisms12050962 (PMC11124045; doi:10.3390/microorganisms12050962)
Supplement: Supplementary file 1 [file microorganisms-12-00962-s001.zip › microorganisms-2953404-supplementary.pdf]

Table S1. Number of avian species collected in Pantanal wetland in the states of Mato Grosso and Mato Grosso do Sul, central-western Brazil.

Scientific name	Common name	Number of sampled birds
Agelaioides badius	Grayish baywing	5
Agelasticus cyanopus	Unicolored blackbird	3
Antilophia galeata	Helmeted manakin	1
Arremon flavirostris	Saffron-billed sparrow	7
Arundinicola leucocephala	White-headed marsh tyrant	4
Basileuterus flaveolus	Flavescent warbler	23
Basileuterus hypoleucus	White-bellied warbler	4
Cacicus cela	Yellow-rumped cacique	10
Cacicus solitarius	Solitary cacique	2
Campylorhynchus turdinus	Thrush-like wren	2
Cantorchilus leucotis	Buff-breasted wren	6
Casiornis rufus	Rufous casiornis	1
Cercomacra melanaria	Mato grosso antbird	9
Certhiaxis cinnamomeus	Yellow-chinned spine tail	18
Cnemotriccus fuscatus	Fuscous flycatcher	5
Coereba flaveola	Bananaquit	4
Conirostrum speciosum	Chesnut-vented conebill	1
Coryphospingus cucullatus	Red-crested finch	1
Cranioleuca vulpina	Rusty-backed spinetail	3
Cyanocorax chrysops	Plush-crested jay	1
Cyanocorax cyanomelas	Purplish jay	3
Cyclarhis gujanensis	Rufous-browed peppershrike	1
Dendroplex picus	Straight-billed woodcreeper	6
Donacobius atricapilla	Black-capped donacobius	5
Elaenia albiceps	White-crested elaenia	2
Elaenia spectabilis	Large elaenia	1
Eucometis penicillata	Gray-headed tanager	5
Euscarthmus meloryphus	Fulvous-crowned scrub tyrant	3
Fluvicola albiventer	Black-backed water tyrant	8
Furnarius leucopus	Pale-legged hornero	13
Furnarius rufus	Rufous hornero	20
Hemitriccus margaritaceiventer	Pearly-vented Tody-tyrant	1
Hemitriccus striaticollis	Stripe-necked Tody-tyrant	5
Herpsilochmus longirostris	Large-billed antwren	1
Hylophilus pectoralis	Ashy-headed greenlet	3
Hypocnemoides maculicauda	Band-tailed antbird	7
Icterus cayanensis	Epaulet oriole	2
Icterus croconotus	Orange-backed troupial	1
Legatus leucophaius	Piratic flycatcher	4
Lepidocolaptes angustirostris	Narrow-billed woodcreeper	1
Machetornis rixosa	Cattle tyrant	5
Molothrus oryzivorus	Giant cowbird	1
Myiarchus ferox	Short-crested flycatcher	6
Myiophobus fasciatus	Bran-colored flycatcher	3
Myiozetetes cayanensis	Rusty-margined flycatchter	5
Paroaria capitata	Yellow-billed cardinal	36
Pipra fasciicauda	Band-tailed manakin	7
Pitangus sulphuratus	Great kiskadee	25
Poecilotriccus latirostris	Rusty-fronted Tody-flycatcher	8
Progne tapera	Brown-chested martin	1
Pseudoseisura unirufa	Grey-crested cacholote	8
Ramphocelus carbo	Silver-beaked tanager	101
Saltator coerulescens	Bluish-gray saltator	18

Sicalis flaveola	Saffron finch	2
Sporophila angolensis	Chesnut-belliend finch	9
Sporophila coerulescens	Double-collared seedeater	1
Sporophila collaris	Rusty-collared seedeater	11
Sporophila lineola	Lined seedeater	1
Stelgidopteryx ruficollis	Southern roughed-wing swallow	3
Synallaxis albilora	White-lored spinetail	10
Taraba major	Great antshrike	4
Thraupis palmarum	Palm tanager	1
Thraupis sayaca	Sayaca tanager	3
Thryothorus genibarbis	Moustached wren	1
Todirostrum cinereum	Common-Tody flycatcher	2
Turdus amaurochalinus	Creamy-bellied thrush	3
Turdus hauxwelli	Hauxwell's thrush	1
Turdus leucomelas	Pale-breasted thrush	8
Turdus rufiventris	Rufous-bellied thrush	7
Tyrannus melancholicus	Tropical kingbird	3
Vireo olivaceus	red-eyed vireo	1
Volatinia jacarina	Blue-black grassquit	3
**Total**		500

Table S2. Conventional, nested and quantitative real-time PCR assays used in screening and molecular characterization for Anaplasmataceae agents targeting the 16S RNA, *groEL, dsb, gltA, sodB, omp-1, rpoB, ftsZ,* and *sucA* genes and intergenic region 23S-5S (ITS).

Agent	Target gene	Primer sequences (5′-3′)	PCR product size (pb)	Thermal protocol	Reference
***Ehrlichia* spp./ *Anaplasma* spp.**	16S rRNA	EHR16SD GGTCCYACAGAAGTCC EHRSR TAGCACTCATCGTTTACAGC	345	95°C for 5 minutes; 40 cycles: 94°C for 1 minute, 54°C for 30 seconds and 72°C for 30 seconds and final extension 72°C for 5 minutes	[44]
***Ehrlichia* spp.**	*dsb*	dsb-330 GATGATGTCTGAAGATATGAA ACAAAT dsb-728 CTGCTCGTCTATTTTACTTCTTA AAGT	409	95°C for 2 minutes; 50 cycles: 95°C for 15 seconds, 58°C for 30 and 72°C for 30 seconds and final extension 72°C for 5 minutes	[45]
***Anaplasma* spp.**	16S rRNA	AnaplsppF, AGAAGAAGTCCCGGCAAACT AnaplR 3GAGACGACTTTTACGGATTAG CTC	800	94°C for 3 minutes; 30 cycles: 94°C for 30 seconds, 50°C for 30 and 72°C for 1 minute and final extension 72°C for 10 minutes	[50]
***Anaplasma* spp./*Ehrlichia* spp.**	16S rRNA	AE1-F AAGCTTAACACATGCAAGTCG AA AE1-R AGTCACTGACCCAACCTTAAA TG	1406	94°C for 3 minutes; 35 cycles: 94°C for 30 seconds, 59°C for 30 seconds and 72°C for	[20]

				1 minute and final extension 72°C for 5 minutes	
Ehrlichia **spp.**	*omp-1*	conP28-F1<br>AT(C/T)AGTG(G/C)AAA(AG)TA(T/C)(A/G)T (G/A)CCAA<br>conP28-R1<br>TTA(G/A)AA(A/G)G(C/T)AAA(C/T) CT(T/G)CCTCC<br>conP28-F2<br>CAATGG(A/G)(T/A)GG(T/C)CC(A/C)AGA (A/G)TAG<br>conP28-R2<br>TTCC(T/C)TG (A/G)TA(A/G)G(A/C)AA(T/G)TTT AGG	700	94°C for 3 minutes;<br>30 cycles: 94°C for 1 minute, 50°C for 1 minute and 72°C for 1 minute and final extension 72°C for 5 minutes	[49]
Ehrlichia **spp.**	*sodB*	sodbEhr1600-F<br>ATGTTTACTTTACCTGAACTTCCATATC<br>sodbEhrl600-R<br>ATCTTTGAGCTGCAAAATCCCAATT	600	94°C for 3 minutes;<br>55 cycles: 94°C for 10 seconds, 58°C for 10 seconds and 72°C for 15 seconds, extension 72°C for 30 seconds and final extension 72°C for 5 minutes	[48]
Anaplasma **spp./*Ehrlichia*** **spp.**	*gltA*	F4b<br>CCGGGTTTTATGTCTACTGC<br>Rb1<br>CGATGACCAAAACCCAT<br>EHR-CS136F<br>TTYATGTCYACTGCTGCKTG<br>EHR-778R<br>GCNCCMCCATGMGCTGG	800<br><br><br>650	95°C for 5 minutes;<br>40 cycles: 95°C for 30 seconds, 55°C for 30 seconds and 72°C for 1 minutes and final extension 72°C for 10 minutes<br><br>95°C for 5 minutes;<br>34 cycles: 95°C for 30 seconds, 55°C for 30 seconds and 72°C for 1 minutes and final extension 72°C for 10 minutes	[47]
Ehrlichia **spp.**	16S rRNA	Eh_16S21F1<br>GGCTCAGAACGAACGCTGG<br>Eh-16S 1494 R1<br>AGCCGCAGGTTCACCTACA<br>EH 16S 31F2<br>GAACGCTGGCGGCAAGCC<br>EH 16S 1467 R2<br>GTTACGACTTCACCMTAGTCA	1474<br><br><br>1437	94°C for 3 minutes;<br>45 cycles: 94°C for 30 seconds, 55°C for 1 minute and 72°C for 1 minute and final extension 72°C for 10 minutes.<br>* Same thermal conditions for the second reaction.	[46]
Ehrlichia **spp.**	*gltA*	Eh_gltA112 F1<br>GGRRTRTTAACTTATGATCCAGG<br>Eh_gltA686 R1<br>GCATTYTGYTCATGATCAGCATG<br>Eh_gltA137 F2<br>TTATGTCTACTGCTGCTTGTGA	575		[46]

		Eh_gltA614 R2<br>TARGAAGAAAYRTCAAACATCATATG	478		
***Ehrlichia* spp.**	*rpoB*	Eh_rpoB241 F1<br>AGTTATAGTATTGGTGARCCRCA<br>Eh_rpoB821 R1<br>ARYCTAACWCCYCTRAAYCTATC<br>Eh_rpoB305 F2<br>CTGTWCCTATACGTATAGTKYTGCG<br>Eh_rpoB623 R2<br>TCTARCCAKGAWCCYCTRTARGG	581<br><br><br><br>319	94°C for 3 minutes; 45 cycles: 94°C for 30 seconds, 55°C for 1 minute and 72°C for 1 minute and final extension 72°C for 10 minutes.<br>* Same thermal conditions for the second reaction.	[46]
***Ehrlichia* spp.**	*ftsZ*	Eh_ftsZ242 F1<br>GTARAGGWGCWGCWGAAGARTCAA<br>Eh_ftsZ703 R1<br>CWGCTTCTCCTGTRCCCATCAT<br>Eh_ftsZ313 F2<br>ACTGCYGGAATGGGTGGWGGA<br>Eh_ftsZ679 R2<br>TTTRCCCATYTCRCTCATTATTGC	462<br><br><br><br>367	94°C for 3 minutes; 45 cycles: 94°C for 30 seconds, 55°C for 1 minute and 72°C for 1 minute and final extension 72°C for 10 minutes.<br>* Same thermal conditions for the second reaction.	[46]
***Ehrlichia* spp.**	*groEL*	Eh_groEL64 F1<br>TTRGAAGAYGCWGTAGGATGYAC<br>Eh_groEL593 R1<br>CCWCKRTCAAAYTGCATRCCATC<br>Eh_groEL107 F2<br>CYGTAGCWATTRGYAARYCYTATGG<br>Eh_groEL341 R2<br>CWNAYAATATCTGCHCCAGCAGC	530<br><br>235	94°C for 3 minutes; 45 cycles: 94°C for 30 seconds, 55°C for 1 minute and 72°C for 1 minute and final extension 72°C for 10 minutes.<br>* Same thermal conditions for the second reaction.	[46]
***Ehrlichia* spp.**	*groEL*	Ehrli-gro67F<br>GAAGATGCWGTWGGWTGTACKGC<br>Ehrli-gro776R<br>AGMGCTTCWCCTTCWACRTCYTC<br>Ehrli-gro217F<br>ATTACTCAGAGTGCTTCTCARTG<br>Ehrli-gro581R<br>TGCATACCRTCAGTYTTTTCAAC	710<br><br>365	94°C for 3 minutes; 45 cycles: 94°C for 30 seconds, 55°C for 1 minute and 72°C for 1 minute final extension 72°C for 10 minutes<br>* Same thermal conditions for the second reaction.	[46, 50]

***Anaplasma* spp.**	16S rRNA	16SF GCGATTTTAGAGTGYGGAGATTG 16SR TACAATACCGGAGTAAAAGTCAA	1133	94°C for 3 minutes; 40 cycles: 94°C for 30 seconds, 56°C for 1 minute and 72°C for 1 minute. final extension 72°C for 10 minutes	[52, 53]
***Anaplasma* spp.**	16 S rRNA	AE4-Fw GTACCYAYAGAAGAAGTCCCG GCA AE-Rv RCACCAGCTTCGAGTTAAGCCA AT GE2F2 GTTAGTGGCAGACGGGTGAGT	800	98°C for 3 minutes; 40 cycles: 98°C for 55 seconds, 57.5°C for 20 seconds and 72°C for 40 seconds final extension 72°C for 5 minutes<br><br>* Same thermal conditions for the second reaction.	[54, 44, 55]
***Anaplasma* spp.**	*groEL*	GROESL1F TATAGCTAGCATAATTACCCAGAGC GROESL1R GGTTAGTTCTGCTTTCGATGC GROESL2F TTATGTCTATGCGCCCGTG GROESL2R CGGACCTTGCCACATTTT	842<br><br>339	94°C for 3 minutes; 30 cycles: 94°C for 30 seconds, 55°C for 30 seconds and 72°C for 1.5 minutes final extension 72°C for 10 minutes<br><br>2ª reação<br><br>94°C for 3 minutes; 30 cycles: 94°C for 30 seconds, 55°C for 30 seconds and 72°C for 1 minute final extension 72°C for 10 minutes	[57]
***Anaplasma* spp.**	ITS 23S-5S	ITSiF ATACCTCTGGTGTACCAGTTG<br><br>ITSiR TTAACTT- CCGGGTTCGGAATG	300	94°C for 2 minutes; 35 cycles: 94°C for 30 seconds, 58°C for 30 seconds and 72°C for 1 minute final extension 72°C for 5 minutes	[56]
***'Candidatus* Allocryptoplasma spp.'**	*sucA*	Crypto_sucA_F1 GTTATGGGNTTTGAGTAYGG Crypto_sucA_R2 GGGCTCTTCYTGRCACCA	636	93°C for 3 minutes; 35 cycles: 93°C for 30 seconds, 52°C For 1 minute and 72°C for 1 minute final extension 72°C for 5 minutes	[4]
***'Candidatus* Allocryptoplasma spp.'**	*groEL*	Crypto_GroEL_F1 CCTTCYTCAACAGCAGCYCTAG Crypto_GroEL_R2 ACNGTTGAAGARAGTAARGG	713		

Table S3. Avian DNA samples positive in the multiplex quantitative (q) real-time qPCR for *Anaplasma* spp. and *Ehrlichia* spp. based on the *groEL* gene.

Sample ID	Avian species	Sampling site	State	Agent detected	Cq
**4 BAP413**	*Certhiaxis cinnamomeus*	Nossa Senhora do Livramento	Mato Grosso	*Anaplasma* spp.	38.32
**51SL 025**	*Eucometis penicillata*	Santo Antonio de Leverger	Mato Grosso	*Anaplasma* spp.	38.30
**70 BEP393**	*Thraupis sayaca*	Corumbá	Mato Grosso do Sul	*Anaplasma* spp.	37.78
**78 F002**	*Leptotila verreauxi*	Santo Antonio de Leverger	Mato Grosso	*Anaplasma* spp.	37.61
**81 BAP435**	*Cantorchilus leucotis*	Nossa Senhora do Livramento	Mato Grosso	*Anaplasma* spp.	36.89
**167 BEP304**	*Ramphocelus carbo*	Corumbá	Mato Grosso do Sul	*Anaplasma* spp.	38.68
**191 BEP340**	*Tigrisoma lineatum*	Corumbá	Mato Grosso do Sul	*Anaplasma* spp.	39.22
**208 BAP04**	*Ramphocelus carbo*	Poconé	Mato Grosso	*Anaplasma* spp.	37.79
**232 BAP87**	*Legatus leucophaius*	Poconé	Mato Grosso	*Anaplasma* spp.	37.95
**244 BAP112**	*Cacicus cela*	Poconé	Mato Grosso	*Anaplasma* spp.	28.94
**264 BAP123**	*Ramphocelus carbo*	Poconé	Mato Grosso	*Anaplasma* spp.	39.24
**266 BAP65**	*Sporophila angolensis*	Poconé	Mato Grosso	*Anaplasma* spp.	39.24
**281 BAP118**	*Saltator coerulescens*	Poconé	Mato Grosso	*Anaplasma* spp.	38.50
**286 BAP42**	*Ramphocelus carbo*	Poconé	Mato Grosso	*Anaplasma* spp.	35.73
**288 BAP128**	*Ramphocelus carbo*	Poconé	Mato Grosso	*Anaplasma* spp.	38.22
**299 BAP15**	*Certhiaxis cinnamomeus*	Poconé	Mato Grosso	*Ehrlichia* spp	37.31

**318 BAP254**	*Furnarius rufus*	Nossa Senhora do Livramento	Mato Grosso	*Ehrlichia* spp	37.52
**332 BAP335**	*Pitangus sulphuratus*	Nossa Senhora do Livramento	Mato Grosso	*Anaplasma* spp.	39.43
**333 BAP257**	*Sporophila collaris*	Nossa Senhora do Livramento	Mato Grosso	*Anaplasma* spp.	37.40
**337 BAP330**	*Agelasticus cyanopus*	Nossa Senhora do Livramento	Mato Grosso	*Anaplasma* spp.	37.14
**350 BAP143**	*Busarellus nigricollis*	Nossa Senhora do Livramento	Mato Grosso	*Anaplasma* spp.	36.53
**356 BAP262**	*Chloroceryle americana*	Nossa Senhora do Livramento	Mato Grosso	*Anaplasma* spp.	36.86
**361 BAP332**	*Agelasticus cyanopus*	Nossa Senhora do Livramento	Mato Grosso	*Anaplasma* spp.	35.50
**365 BAP220**	*Tyrannus melancholicus*	Nossa Senhora do Livramento	Mato Grosso	*Anaplasma* spp.	34.57
**384 BAP139**	*Guira guira*	Nossa Senhora do Livramento	Mato Grosso	*Anaplasma* spp.	35.96
**388 BAP263**	*Paroaria capitata*	Nossa Senhora do Livramento	Mato Grosso	*Anaplasma* spp.	37.21
**400 SL80**	*Icterus cayanensis*	Santo Antonio de Leverger	Mato Grosso	*Anaplasma* spp.	36.68
**403 F14**	*Leptotila verreauxi*	Santo Antonio de Leverger	Mato Grosso	*Anaplasma* spp.	36.82
**417 F73**	*Ramphocelus carbo*	Santo Antonio de Leverger	Mato Grosso	*Anaplasma* spp.	38.20
**423 F40**	*Leptotila verreauxi*	Santo Antonio de Leverger	Mato Grosso	*Anaplasma* spp.	38.12
**436 F86**	*Ramphocelus carbo*	Santo Antonio de Leverger	Mato Grosso	*Anaplasma* spp.	36.89
**461 F56**	*Pitangus sulphuratus*	Santo Antonio de Leverger	Mato Grosso	*Anaplasma* spp.	36
**463 F67**	*Pipra fasciicauda*	Santo Antonio de Leverger	Mato Grosso	*Anaplasma* spp.	36.53
**480 SL32**	*Cranioleuca vulpina*	Santo Antonio de Leverger	Mato Grosso	*Anaplasma* spp.	34.66

**487 SL42**	*Phaethornis nattereri*	Santo Antonio de Leverger	Mato Grosso	*Anaplasma* spp.	35.82
**489 F27**	*Basileuterus flaveolus*	Santo Antonio de Leverger	Mato Grosso	*Anaplasma* spp.	39.05
**496 F42**	*Ramphocelus carbo*	Santo Antonio de Leverger	Mato Grosso	*Anaplasma* spp.	33.46
**498 SL57**	*Dendroplex picus*	Santo Antonio de Leverger	Mato Grosso	*Anaplasma* spp.	33.47
				Ehrlichia spp.	33.24
**516 F50**	*Ramphocelus carbo*	Santo Antonio de Leverger	Mato Grosso	*Anaplasma* spp.	33.40
				Ehrlichia spp.	37.89

Table S4. BLAST analyses results obtained for 16S rRNA, *dsb*, and ITS sequences obtained in PCR protocols for Anaplasmataceae agents from avian blood samples from the Brazilian Pantanal.

**Sample ID**	**Bird species/ Locality**	**Molecular marker**	**Sequence size (bp)**	**Query cover (%)**	**E-value**	**Identity (%)**	**Best Match**	**Host**	**Accession number**	**Country**	**Reference of PCR protocol used**
**264**	*Ramphocelus carbo* (BAP123), Poconé Mato Grosso.	16S rRNA	209	100	1.00 E-96	98.58	*Ehrlichia* spp.	*Amblyomma sculptum*	MT514732	Brazil	[44]
**292**	*Elaenia albiceps (BAP72)* Poconé, Mato Grosso	16S rRNA	249	100	2.00 E-139	99.65	*Ehrlichia* spp.	*Erinaceus amurensis*	MH879869	China	[44]
**441**	*Pitangus sulphuratus* (F10) Santo Antonio de Leverger, Mato Grosso	16S rRNA	205	100	1.00 E-96	99.51	*Anaplasma* spp.	*Amblyomma hebraeum*	MG351089	Eswatini	[44]

**444**	*Ramphocelus carbo* (F68) Santo Antonio de Leverger, Mato Grosso	16S rRNA	281	100	2.00 E^{-139}	100	*Ehrlichia* spp.	*Haemaphysalis elliptica*	MZ351092	Eswatini	[44]
**330**	*Crax fasciolata* (BAP146) Nossa Senhora do Livramento, Mato Grosso	16S rRNA	269	100	2.00 E^{-128}	98.88	*Anaplasma* spp.	*Amblyomma dissimile*	MG437272	Brazil	[44]
				100	3.00 E^{-127}	98.51	*Candidatus* Allocryptoplasma spp.	*Lacerta viridis*	MG924904	Slovakia	
**330**	*Crax fasciolata* (BAP146) Nossa Senhora do Livramento, Mato Grosso	16S rRNA	884	100	0	98.6	‘*Candidatus* Alloryptoplasma sp.’	*Lacerta viridis*	MG924904	Slovakia	[54]
				99	0	98.98	*Anaplasma* sp.	*Haemaphysalis parmata*	OQ092428	Uganda	
**330**	*Crax fasciolata* (BAP146) Nossa Senhora do Livramento, Mato Grosso	16S rRNA	908	100	0	99.56	‘*Candidatus* Alloryptoplasma sp.’	*Amblyomma coelebs*	OQ724839	French Guyana	
				99	0	99.56	‘*Candidatus* Alloryptoplasma sp.’	*Amblyomma coelebs*	OR854270	French Guyana	Consensus sequence

**341**	*Furnarius leucopus* (BAP428) Nossa Senhora do Livramento, Mato Grosso	16S rRNA	832	100	0	98.9	*Anaplasma* sp.	*Haemaphysalis parmata*	OQ092428	Uganda	[54]
				100	0	99.3	*Anaplasma* sp.	*Amblyoma tholloni*	OQ092427	Uganda	
**345**	*Aurindinicola leucocephala* (BAP267) Nossa Senhora do Livramento, Mato Grosso	16S rRNA	832	100	0	97.4	*Ehrlichia* sp.	*Dasyurus geoffroii*	MW633161	Australia	[54]
				100	0	97.3	*Ehrlichia* sp.	*Ixodes auritulus*	MW628650	Uruguay	
**389**	*Ageslastycus cyanopus* (BAP289) Nossa Senhora do Livramento, Mato Grosso	*dsb*	401	99	0	99	*Ehrlichia minasensis*	*Bradypus variegatus*	MT212414	Rondônia, Brazil	[54]
**418**	*Basileuterus flaveolus* (F54) Poconé, Mato Grosso	*dsb*	402	100	0	99.25	*E. minasensis*	*B. variegatus*	MT212414	Rondônia, Brazil	[54]

**330**	*Crax fasciolata* (BAP146) Nossa Senhora do Livramento, Mato Grosso	23S-5S rRNA (ITS)	261	100<br>73	$6E^{-84}$<br>$1E^{-61}$	89.53<br>90.10	*Anaplasma phagocytophilum*<br>*Anaplasma marginale*	*Sceloporus occidentalis*<br>*Bos taurus*	JF487930<br>CP023731	USA<br>Brazil	[56]
**497**	*Saltator coerulescens* (F58) Santo Antonio de Leverger, Mato Grosso	23S-5S rRNA (ITS)	270	77<br>88	$5E^{-55}$<br>$5E^{-55}$	86.19<br>86.19	*A. phagocytophilum*<br>*A. phagocytophilum*	*Ovis aries*<br>*Homo sapiens*	CP015376<br>CP035303	Norway<br>South Korea	[56]
**507**	*Basileuterus flaveolus* (F12) Nossa Senhora do Livramento, Mato Grosso	23S-5S rRNA (ITS)	267	89<br>100	$4E^{-101}$<br>$33E^{-77}$	91<br>83.84	*A. phagocytophilum*<br>*A. marginale*	*S. occidentalis*<br>*B. taurus*	JF487930<br>CP023731	USA<br>Brazil	[56]
